# Prospective associations between peer functioning and social anxiety in adolescents: A systematic review and meta-analysis

**DOI:** 10.1016/j.jad.2020.10.055

**Published:** 2021-01-15

**Authors:** Kenny Chiu, David M. Clark, Eleanor Leigh

**Affiliations:** a Department of Psychology, Institute of Psychiatry, Psychology & Neuroscience, King's College London, London, UK; b Department of Experimental Psychology, University of Oxford, Oxford, UK; c Department of Experimental Psychology, University of Oxford, Oxford, UK

**Keywords:** Adolescents, Friendship Quality, Peer Acceptance, Peer Rejection, Peer Victimization, Prospective, Social Anxiety

## Abstract

**Background:**

During adolescence, peer relationships take precedence and there is a normative increase in social anxiety. Although prospective studies have suggested peer functioning and social anxiety can influence each other, their findings have not been examined systematically. We performed a systematic review and meta-analysis of prospective studies to examine the bidirectional relationship between peer functioning and social anxiety in adolescence.

**Methods:**

EMBASE, PsycINFO, Medline, and PubMed were searched to identify relevant articles. Meta-analysis was conducted to examine the mean effect sizes of prospective associations between social anxiety and four dimensions of peer functioning. Moderator analysis was performed, with age, gender, time interval between baseline and follow-up assessment, and publication year as moderators.

**Results:**

Meta-analyses of 23 studies showed that friendship quality (r =-.11), peer rejection (r =-.06), and peer victimization (r =.23) were each associated with later social anxiety, but peer acceptance was not (r =-.11). Social anxiety at baseline was associated with prospective levels of friendship quality (r =-.11), peer rejection (r=.09), and peer victimization (r =.17), but not peer acceptance (r =-.14). Age moderated the association between friendship quality and prospective social anxiety. Other moderator effects were statistically non-significant.

**Limitations:**

Limitations include different classifications of peer functioning, the use of self-report measures, heterogeneity between studies, and underrepresentation of clinical samples.

**Conclusions:**

A significant bidirectional association was found with social anxiety across three dimensions of peer functioning. Psychological prevention and intervention targeting peer functioning and social anxiety are indicated.

## Highlights

• This systematic review and meta-analysis examined adolescent peer functioning and social anxiety.
• Friendship quality, peer rejection, and peer victimization were associated with later social anxiety.
• Social anxiety was associated with later friendship quality, peer rejection, and peer victimization.
• Peer acceptance was not associated with social anxiety.
• Prevention and intervention targeting peer functioning and social anxiety are indicated.

## 1 Introduction

Adolescence is a time when there is a normative increase in social anxiety symptoms (Westenberg et al., 2007) and when Social Anxiety Disorder (SAD; American Psychiatric Association, 2013) typically first occurs (Kessler et al., 2005). It is also a period of social reorientation (Nelson et al., 2005): children typically move to secondary school and spend more time with their peers than at any other time of life (Furman & Buhrmester, 1992). The ability to form and maintain friendships and integrate with one's peer group (peer functioning; Prinstein et al., 2000) becomes crucial. Previous studies have examined the role of peer functioning in maintaining social anxiety in non-clinical and clinical adolescent samples (de Lijster et al., 2018; Epkins & Heckler, 2011). It is important to understand the interrelationship between peer functioning and social anxiety because youth who develop extreme and persistent fear and avoidance of social situations (i.e. SAD) are at greater risk of developing subsequent depression (Beesdo et al., 2007), alcohol use (Black et al., 2016), and other types of anxiety disorders (Wittchen et al., 1999).

Important aspects of peer functioning include: friendship quality, peer acceptance, peer rejection, and peer victimization (Prinstein & Giletta, 2020). These four aspects of peer functioning have been assessed with different measures, but each of them has been found to be associated with important psychosocial outcomes. Friendship quality has been assessed using self-report measures such as the Friendship Quality Questionnaire (Parker & Asher, 1993), and can be further divided into positive and negative dimensions. Positive friendship quality encompasses the role of friends in meeting adolescents’ needs for companionship (e.g. “always sit together at lunch”), intimacy (e.g. “always telling each other about our problems”), and support (e.g. “helps me with things so I can get done quicker”). Items assessing negative friendship quality, such as conflict and betrayal (e.g. “get mad at each other a lot”), have also been included in these scales. Empirical studies examining friendship quality have found that it is prospectively associated with both physical and mental health outcomes. Positive friendship quality has been found to be associated with better physical health outcomes in adulthood (Allen et al., 2015), a lower risk of suicide attempt (Tuisku et al., 2014), and a lower level of psychological distress (Dion et al., 2016). Negative friendship quality has been observed to be associated with later depressive symptoms (Schwartz-Mette et al., 2020).

Peer acceptance, which refers to the degree to which an individual is accepted by peers, has been assessed in some studies by asking participants to list the names of their good friends at school (Lessard & Juvonen, 2018). In other studies participants were instructed to rate Likert scale items on perceived peer acceptance (e.g. “many classmates like to do things together with me”; Barzeva et al., 2019). Peer acceptance has been reported to predict a range of outcomes, including a higher level of academic achievement, better self-esteem, and lower loneliness (Gallardo et al., 2016; Kingery et al., 2011).

Peer rejection, which refers to the degree to which an individual is disliked by peers, has been assessed by asking respondents to nominate the peers they liked the least (Sentse et al., 2017) or report the frequency of perceived rejection (Vernberg et al., 1992). Higher levels of peer rejection have been found to predict an increase in physical health problems, more aggression towards romantic partners, and more frequent peer victimization (Brendgen & Vitaro, 2008; Demol et al., 2020; Schacter et al., 2019).

Peer victimization refers to the repeated experiences of aggressive acts from peers (Olweus, 2001). Peer victimization may be overt, “how often are you being hit, kicked, locked up?”, relational, “how often do children say mean things to you?”, or reputational, “other teens told lies about you to make other teens not like you anymore”. Peer victimization is assessed by asking participants to report their own experiences or nominate their peers who are victimized (“which three classmates are often victimized by other children?”) (e.g. Mulder et al., 2017). Studies have consistently found that peer victimization is associated with poorer physical health and a heightened risk for anxiety disorders and depression in adulthood (Bowes et al., 2015; Copeland et al., 2013; Hager & Leadbeater, 2016; Stapinski et al., 2014).

Although peer functioning is associated with a number of psychosocial and physical health outcomes, its relationship with social anxiety is less clear. It has been suggested that peer functioning and social anxiety may be reciprocally linked (Alden & Taylor, 2004), with negative peer interactions reinforcing social fears and avoidance (Epkins & Heckler, 2011; Sentse et al., 2017) and also the interpersonal behaviors of socially anxious individuals inadvertently evoking unfriendly reactions from peers, leading to peer rejection or peer victimization (Leigh & Clark, 2018). Evidence has accumulated in support of this assertion. A systematic review by de Lijster et al. (2018) examined the social and academic functioning in adolescents with a diagnosed anxiety disorder and those without a clinical diagnosis. In this review they examined social competence, interpersonal relationships, peer victimization, and social acceptance. They concluded that adolescents with SAD tend to experience more peer victimization, lower peer acceptance, and higher levels of loneliness. However, all of the studies with SAD samples that this review was based on adopted a cross-sectional design. The use of a cross-sectional design precludes us from drawing causal inferences, instead a longitudinal study design is recommended to test reciprocal processes (Sameroff & Mackenzie, 2003). A narrative review by Epkins and Heckler (2011) synthesized findings from non-clinical and clinical studies examining the relationships between peer functioning and social anxiety in young people. They examined different aspects of peer functioning, including friendship quality, peer acceptance, peer rejection, peer victimization, loneliness, and social skill deficits. Regarding peer acceptance and rejection and social anxiety, the authors found support for bidirectional associations. Findings were mixed from the two prospective studies on peer victimization (Siegel et al., 2009; Storch et al., 2005), whilst only one prospective study had examined the association between friendship quality and social anxiety (Vernberg et al., 1992). As more prospective studies have been published over the past decade, an up-to-date review is needed to evaluate them and to quantify the bidirectional relationships.

Using a systematic review and meta-analysis, the current study aimed to examine evidence for a reciprocal link between peer functioning and social anxiety in adolescents aged 10–19 years. Studies of youth aged 10–19 years old were included because this age range covers early adolescence (10–14 years old) and late adolescence (15–19 years old) as defined by WHO (2016). Only a few prospective studies used a non-continuous measure of social anxiety, and therefore this review focused on studies that used a continuous measure of social anxiety. As peer functioning is multi-faceted, four dimensions of peer functioning were examined: friendship quality, peer acceptance, peer rejection, and peer victimization. These dimensions are distinctive from each other as they have been assessed using different measures in previous studies (e.g. Grills-Taquechel et al., 2010; Tillfors et al., 2012; Vernberg et al., 1992). Positive and negative friendship qualities have been examined either as an unitary construct (Mak et al., 2018) or two distinctive constructs (Tillfors et al., 2012). Therefore, studies examining positive and negative friendship qualities were reviewed together then separately. In this study we also examined the following study characteristics as potential moderators in the prospective associations: age, gender, time interval between baseline and follow-up assessment, and publication year.

## 2 Methods

### 2.1 Search Strategy

The review conforms to the PRISMA statement (see Supplementary Materials, Appendix 1 for the PRISMA checklist; Moher et al., 2009). Studies published between 2 October 1967 to 9 May 2020 were retrieved from EMBASE, PsycINFO, Medline, and PubMed. The earliest relevant article was published in 1967. The following key words were used in extracting relevant articles: (young* or youth or adoles*) and (social anxi* or social phobi*) and (peer* or friends* or relations* or interpersonal* or victimis* or bully* or harass* or intimidat* or delinquen*). Duplicates were removed. Titles and abstracts of studies were screened based on the eligibility criteria. Reference lists of included studies were screened to identify relevant articles. A full search electronic search strategy is provided for the Medline database in the Supplementary Materials (Appendix 2).

### 2.2 Study Selection

Studies were included if they (1) involved participants who were aged 10–19 years at the first assessment time point, (2) applied at least one continuous measure for social anxiety and for a dimension of peer functioning, (3) measured the prospective associations between peer functioning and social anxiety, and (4) were published in English language, peer-reviewed, and indexed scientific journals. Studies were excluded if (5) they were primarily interested in studying individuals with neurodevelopmental conditions or physical health conditions. The reason is that findings from these studies may have limited generalisability to the general population. Studies were also excluded if (6) the same sample and measures were used in another study already identified in this review, or (7) the study was a review article, a conference abstract or paper, or a research dissertation.

### 2.3 Quality Assessment

Study quality was assessed using the Quality Assessment Tool for Observational Cohort and Cross-Sectional Studies (National Heart, Lung & Blood Institute, 2014). This 14-question checklist has been suggested as a suitable tool for assessing important characteristics of prospective cohort studies (Ma et al., 2020). The 14 questions were related to (1) research objective, (2) study population, (3) participation rate, (4) participant recruitment and application of eligibility criteria, (5) sample size justification, (6) exposure assessed prior to outcome measurement, (7) time interval between baseline assessment and follow-up assessment, (8) different levels of the exposure measure, (9) a clear definition of the exposure measure, (10) repeated exposure measurement, (11) a clear definition of the outcome measure, (12) blinding of outcome assessor(s), (13) follow-up rate, and (14) statistical control for confounding variables. A total quality score was derived by summing up all the yes-no responses (0 = ‘no’, 1 = ‘yes’). One of three overall quality ratings was assigned to each study (10 < ‘poor’, 10 = ‘fair’, 10 > ‘good’). Studies were assessed independently by two assessors (KC and JH). Any discrepancies in scorings were discussed and resolved, and discussion outcomes were recorded electronically. The interclass correlation coefficient between the two assessors was .80 with a 95% confidence interval of .55 – .91 (F (24, 24) = 5.07, p < .001).

### 2.4 Data Extraction

The following information was extracted in duplicate: (1) sample size, (2) age range and/or average age, (3) proportion of female participants, (4) number of months between the first (T1) and the second (T2) assessment points, (5) country where the study was conducted, (6), publication year, (7) type of social anxiety measure, (8) social anxiety informant (self- or peer-report), (9) type of peer functioning measure, and (10) peer functioning informant (self- or peer-report), and (11) the effect size of the association between T1 peer functioning and T2 social anxiety, and (12) the effect size of the association between T1 social anxiety and T2 peer functioning.

### 2.5 Data analysis

Pearson's correlation coefficient (r) was chosen as the effect size because it is commonly reported in observational studies. For studies that did not report r, standardized regression coefficients were converted to r as suggested by Peterson and Brown (2005). Odds ratios were transformed to r following the recommendations by Borenstein, Hedges, Higgins, and Rothstein (2009). When studies reported effect sizes for girls and boys separately, effect sizes were combined using the formula suggested by Borenstein, Hedges, Higgins, and Rothstein (2009). When studies used two or more questionnaire measures for one dimension of peer functioning or social anxiety, effect sizes obtained from each measure were averaged.

Meta-analyses were conducted using RStudio (R Core Team, 2019) and the metafor package in R (Viechtbauer, 2019). A random-effects meta-analysis model was used because variations in outcomes between studies was expected due to differences in study characteristics (e.g. age of participants, gender, interval). Effect size of each study was converted to Fisher's Z for meta-analysis, and the summary Fisher's Z score was converted back to a summary correlation. Cohen's guidelines (Cohen, 1988) were used to interpret the magnitude of effect sizes (r = .10 ‘small effect’, r = .30 ‘moderate effect’, r = .50 ‘large effect’). The Cochran's Q test and the Higgin's and Thompson's I^2^ test were used to assess the degree of heterogeneity between studies. A statistically significant result from the Cochran's Q test (p < .05) suggests the presence of heterogeneity. A higher I^2^ value indicates a higher degree of heterogeneity (25% = ‘low heterogeneity’, 50% = ‘moderate heterogeneity’, 75% = ‘substantial heterogeneity’; Higgins, Thompson, Deeks, & Altman, 2003). Risk of publication bias across studies was evaluated by inspecting the funnel plots and running the Egger's test (Egger et al., 1997). A significant Egger's test statistic (p < .05) suggests there is substantial asymmetry in the funnel plot, and such asymmetry is indicative of publication bias. A series of meta-regressions were conducted to examine several study characteristics as potential moderators: (1) age (coded as average age of participants), (2) gender (coded as proportion of female participants), (3) interval (coded as number of months between the first and the second data assessment points), and (4) publication year.

## 3 Results

### 3.1 Search results

**Figure 1:**
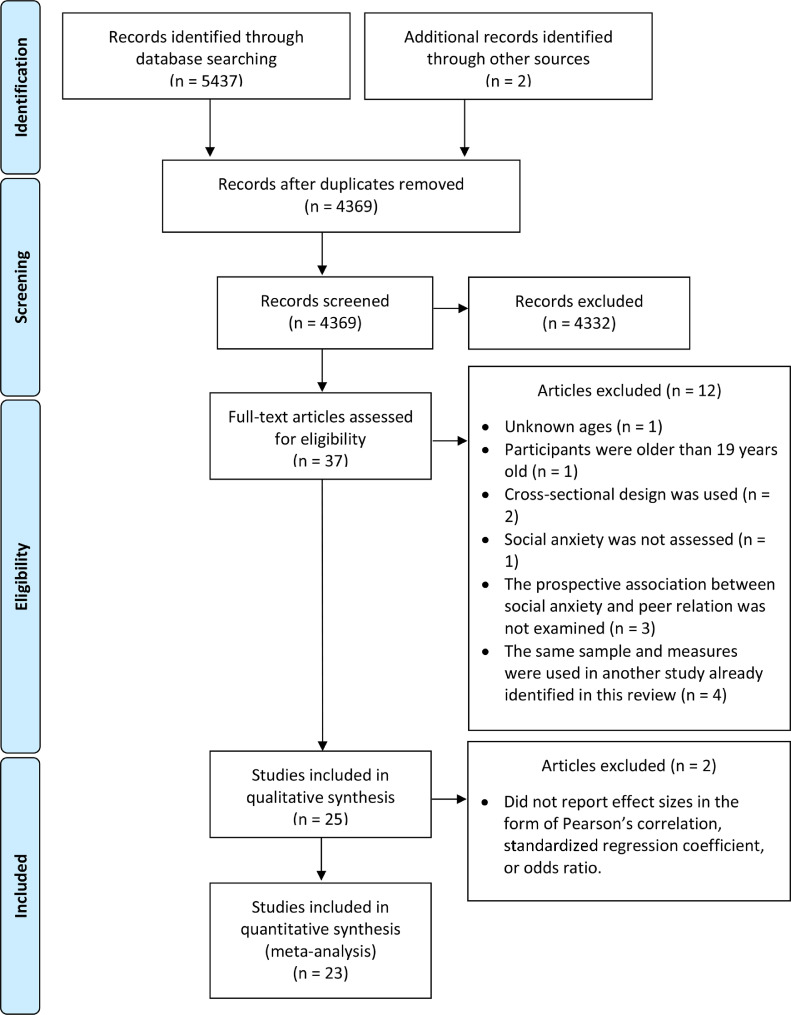
PRISMA Diagram

Figure 1 displays the literature search process using a PRISMA diagram (Moher, Liberati, Tetzlaff, Altman, & The PRISMA Group, 2009). KC performed the initial literature search and screening. Two coders (KC and JH) reviewed 37 articles independently and the inter-rater reliability was excellent (Kappa coefficient = 1.00). After excluding 12 ineligible studies, 25 studies were retained for quality assessment. Two articles were excluded as their data could not be converted to effect sizes suitable for meta-analysis. Therefore, a total number of 23 studies were included in the meta-analysis.

### 3.2 Study characteristics

Table 1, Table 2, Table 3 summarise study characteristics. Sample sizes ranged from 68 to 5991 (M = 1511, SD = 16.51). Participants were between 10 and 19 years (M = 13.18, SD = 2.21). Percentages of female participants ranged from 39% to 64% (M = 51.81, SD = 5.19). Almost all studies recruited participants from schools or local communities, except for one study, which also recruited participants from mental health clinics (Barzeva et al., 2019). In twelve studies the majority of the participants were identified as middle class. Study methodological quality ranged from 9 to 14. Only one study was rated as ‘poor’.

**Table 1:** Summary of Studies Examining the Prospective Associations between Friendship Quality (FQ) and Social Anxiety (SA)

Study	Sample size	Age (range/ mean)	Percentage of female participants	Time interval (months)	Country	SA measure	FQ measure	Effect size: SA to FQ	Effect size: FQ to SA	Quality (range: 0-14)
Vernberg et al. (1992)	68	12–14	44%	2	USA	SASC-R	FI	-.12	n/a	Fair (10)
Grills-Taquechel et al. (2010)	77	11–13	52%	24	USA	MASC	SSSC	-.05	-.10	Good (11)
Tillfors et al. (2012)	1528	12–19	49%	12	Sweden	SPSQ-C	FQQ	-.06	-.10	Good (11)
Biggs et al. (2012)	214	13	50%	2	USA	SASC-R	FI	-.08	n/a	Good (11)
Väänänen et al. (2014)	2070	16	49%	24	Finland	SPIN	PSSS-R	n/a	-.01	Good (11)
Van Zalk and Van Zalk (2015)	2194	10–18	48%	12	Sweden	SPSQ-C	FQQ	-.11	-.03	Good (11)
Cavanaugh and Buehler (2016)	436	11–14	51%	12	USA	SASC-R	FSS and FI	-.11	-.26	Good (12)
Ranta et al. (2016)	3278	18	49%	24	Finland	SPIN	PSSS-R	n/a	-.07	Good (11)
Mak et al. (2018)	687	11	52%	18	USA	SAS-A	FQQ-SF	-.19	-.24	Fair (10)

Notes: n/a = Not available. SASC-R = Social Anxiety Scale for Children-Revised (La Greca & Stone, 1993), MASC = Multidimensional Anxiety Scale for Children (March et al., 1997), SPSQ-C = Social Phobia Screening Questionnaire for Children and Adolescents (Gren-Landell et al., 2009), SPIN = Social Phobia Inventory (Connor et al., 2000), SAS-A = Social Anxiety Scale for Adolescents (La Greca & Lopez, 1998), FI = Friendship Interview (Berndt & Perry, 1986), SSSC = Social Support Scale for Children (Harter, 1986), FQQ = Friendship Quality Questionnaire (Parker & Asher, 1993), FQQ-SF = Friendship Quality Questionnaire-Short Form (Tu et al., 2014), PSSS-R = Perceived Social Support Scale-Revised (Blumenthal et al., 1987), FSS = Friendship Support Scale (Richman & Bowen, 2004).

**Table 2:** Summary of Studies Examining the Prospective Associations between Peer Acceptance (PA) and Social Anxiety (SA)

Study	Sample size	Age (range/ mean)	Percentage of female participants	Time interval (months)	Country	SA measure	PA measure	Effect size:		
SA to PA	Effect size:									
PA to SA	Quality (range: 0-14)									
Grills-Taquechel et al. (2010)	77	11–13	52%	24	USA	MASC	SPPC	-.36	-.29	Good (11)
Tillfors et al. (2012)	1528	12–19	49%	12	Sweden	SPSQ-C	FN	-.04	-.10	Good (11)
Barzeva et al. (2019)	2772	10–12	51%	24	Netherlands	RCADS	SPF	-.09	-.05	Good (11)

Notes: MASC = Multidimensional Anxiety Scale for Children (March et al., 1997), SPSQ-C = Social Phobia Screening Questionnaire for Children and Adolescents (Gren-Landell et al., 2009), RCADS = Revised Children's Anxiety and Depression Scale (Chorpita et al., 2005), SPPC = Self-perception Profile for Children (Harter, 1985), FN = Friendship Nomination (Kerr et al., 2007), SPF = Social Production Function (Ormel et al., 1997).

**Table 3:** Summary of Studies Examining the Prospective Associations between Peer Rejection (PR) and Social Anxiety (SA)

Study	Sample size	Age (range/ mean)	Percentage of female participants	Time interval (months)	Country	SA measure	PR measure	Effect size: SA to PR	Effect size: PR to SA	Quality (range: 0-14)
Vernberg et al. (1992)	68	12–14	44%	2	USA	SASC-R	REQ	-.06	n/a	Fair (10)
Sentse et al. (2017)	5645	14	51%	7	Finland	FNE	FN	-.09	-.09	Fair (10)
Lessard and Juvonen (2018)	5991	11–13	52%	36	USA	SAS-A	FN	n/a	-.03	Good (12)

Notes: FNE = Fear of Negative Evaluation Scale (Garcia-Lopez et al., 2001), SAS-A = Social Anxiety Scale for Adolescents (La Greca & Lopez, 1998), SASC-R = Social Anxiety Scale for Children-Revised (La Greca & Stone, 1993), REQ = Rejection Experiences Questionnaire (Vernberg, 1990), FN = Friendship Nomination (Kerr et al., 2007).

**Table 4:** Summary of Studies Examining the Prospective Associations between Peer Victimization (PV) and Social Anxiety (SA)

Study	Sample size	Age (range/ mean)	Percentage of female participants	Time interval (months)	Country	SA measure	PV measure	Effect size: SA to PV	Effect size: PV to SA	Quality (range: 0-14)
Storch et al. (2005)	144	13–15	64%	12	USA	SAS-A	SEQ	.34	.38	Fair (10)
Siegel et al., (2009)	228	14–19	58%	2	USA	SAS-A	RPEQ	.19	.22	Good (11)
Tillfors et al. (2012)	1528	12–19	49%	12	Sweden	SPSQ-C	PVQ	.10	.13	Good (11)
Loukas and Pasch (2013)	490	10–14	53%	24	USA	SAS-A	SEQ	n/a	.35	Good (11)
Ranta et al. (2013)	2070	15–16	49%	24	Finland	SPIN	PVQ	.13	.14	Good (11)
Acquah et al. (2016)	390	12–13	50%	3	Finland	SAS-C	PVQ	.22	n/a	Poor (9)
Hamilton et al. (2016)	410	12–13	53%	9	USA	MASC	SEQ	.16	.37	Good (11)
Sentse et al. (2017)	5645	14	51%	7	Finland	FNE	OB/VQ	.15	.19	Fair (10)
González-Díez et al. (2017)	550	16–19	56%	6	Spain	SAQ-A30	PRQ	n/a	.16	Good (11)
Mulder et al. (2017)	1649	16–19	55%	6	Netherlands	SAS-K	PNV, OB/VQ	.24	.23	Fair (10)
Calvete et al. (2018)	1328	15.05	45%	6	Spain	SAS-A	PVQ	.26	.33	Good (11)
Barzeva et al. (2019)	2772	10–12	51%	24	Netherlands	RCADS	YSR, CBCL	.14	.12	Good (11)
Chu et al. (2019)	611	11–15	39%	6	China	SCS	TBV	.05	.11	Fair (10)
Irwin et al. (2019)	396	10–13	50%	12	Canada	SAS-A	OB/VQ	n/a	.28	Good (11)

Notes: SASC-R = Social Anxiety Scale for Children-Revised (La Greca & Stone, 1993), SAS-A = Social Anxiety Scale for Adolescents (La Greca & Lopez, 1998), SPSQ-C = Social Phobia Screening Questionnaire for Children and Adolescents (Gren-Landell et al., 2009), SPIN = Social Phobia Inventory (Connor et al., 2000), FNE = Fear of Negative Evaluation Scale (Garcia-Lopez et al., 2001), MASC = Multidimensional Anxiety Scale for Children, SAQ-A30 = The Social Anxiety Questionnaire for Adults (Caballo et al., 2012), SAS-K = Dutch Social Anxiety Scale for Adolescents (Dekking, 1983), RCADS = Revised Children's Anxiety and Depression Scale (Chorpita et al., 2000), SCS = Self-Consciousness Scale (Chu et al., 2019), SEQ = Social Experience Questionnaire-Self Report Form (Crick & Grotpeter, 1995), RPEQ = Revised Peer Experience Questionnaire (de Los Reyes & Prinstein, 2004), PVQ = Peer Victimization Questionnaire (Kaltiala-Heino et al., 1999), PRQ = Peer Relations Questionnaire for Children (Rigby & Slee, 1993), OB/VQ = Olweus Bully/Victim Questionnaire (Olweus, 1986), YSR = Youth Self-Report Depressive/Withdrawn Scale (Achenbach & Rescorla, 2001), CBCL = Child Behavioral Checklist (Achenbach & Rescorla, 2001), TBV = Traditional Bullying Victimization (Li et al., 2012).3.3 Meta-analyses of T1 Peer Functioning and T2 Social Anxiety Data

Nine studies used self-report questionnaires to measure positive and negative qualities of friendship. Positive qualities included companionship (Biggs et al., 2012; Mak et al., 2018; Van Zalk & Van Zalk, 2015; Vernberg et al., 1992), intimacy (Biggs et al., 2012; Mak et al., 2018; Van Zalk & Van Zalk, 2015; Vernberg et al., 1992), and peer support (Cavanaugh & Buehler, 2016; Grills-Taquechel et al., 2010; Ranta et al., 2016; Tillfors et al., 2012; Väänänen et al., 2014). Negative qualities included relational negativity (Tillfors et al., 2012) and conflict (Mak et al., 2018). Three studies examined peer acceptance, with two on perceived peer acceptance (Barzeva et al., 2019; Grills-Taquechel et al., 2010) and one on peer nomination (Tillfors et al., 2012). Three studies examined peer rejection using self-report measures or peer nominations (Lessard & Juvonen, 2018; Sentse et al., 2017; Vernberg et al., 1992). Fourteen studies focused on peer victimization, with nine studies measured its subtypes (e.g. overt, relational, reputational, direct, indirect, emotional). Two studies used peer-report measures to assess peer victimization (Cavanaugh & Buehler, 2016; Mulder et al., 2017).

All studies collected data at least twice, with a time interval of 2–36 months. Almost all studies reported zero-ordered correlations. One study reported partial correlations after adjusting for the effect of age (Tillfors et al., 2012). Some studies reported standardized regression coefficients (Biggs et al., 2012; Grills-Taquechel et al., 2010; Hamilton et al., 2016; Storch et al., 2005), or odds ratios (Ranta et al., 2013, 2016; Väänänen et al., 2014).

#### 3.3.1 Friendship quality

**Figure 2:**
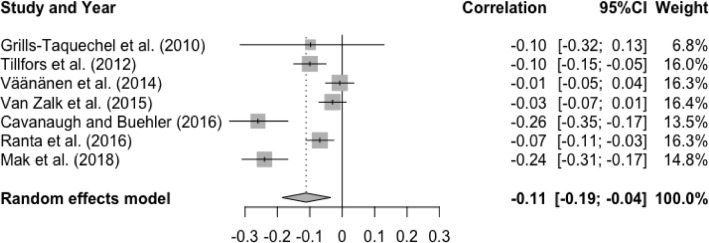
Forest Plot of Correlations between T1 Friendship Quality and T2 Social Anxiety and 95% Confidence Interval for Random Effects Model

The meta-analysis examining the association between T1 friendship quality and T2 social anxiety showed a significant, small effect size, r = -.11, p < .01, 95% CI [-0.19, -0.04], indicating that higher levels of friendship quality at baseline were associated with lower levels of social anxiety at follow-up (See Figure 2). There was a statically significant and substantial degree of heterogeneity, Q(6) = 49.54, p < .0001, I^2^ = 87.9%. Significantly greater and more negative effect sizes were found in younger study samples (Q(1) = 7.71, p < .01), compared to older study samples. There was a non-significant moderator effect of gender (Q(1) = 0.24, p = .62), interval (Q(1) = 0.69, p = .40), and publication year (Q(1) = 1.46, p = .23).

**Figure 3:**
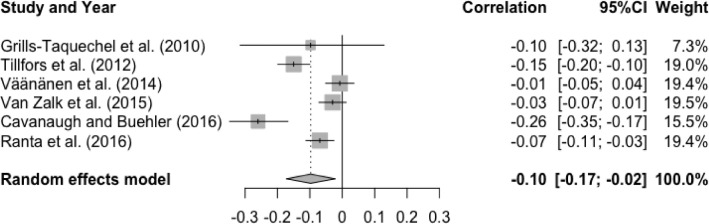
Forest Plot of Correlations between T1 Positive Friendship Quality and T2 Social Anxiety and 95% Confidence Interval for Random Effects Model

The meta-analysis was repeated for positive and negative friendship qualities to see if they were differentially linked to social anxiety. Analysis of six studies showed a significant small effect size for the association between T1 positive friendship quality and T2 social anxiety, r = -.10, p < .05, 95% CI [-0.17, -0.02], indicating that higher levels of positive friendship quality at baseline were associated with lower levels of social anxiety at follow-up (See Figure 3). There was a significant and substantial degree of heterogeneity, Q(5) = 37.39, p < .0001, I^2^ = 86.6%. All the moderator effects were non-significant: age (Q(1) = 2.40, p = .12), gender (Q(1) = 0.55, p = .46), interval (Q(1) = 1.53, p = .22), and publication year (Q(1) = 0.01, p = .91). The analysis was not repeated for negative friendship quality due to small number of available studies (n = 1, Tillfors et al., 2012).

#### 3.3.2 Peer acceptance

**Figure 4:**
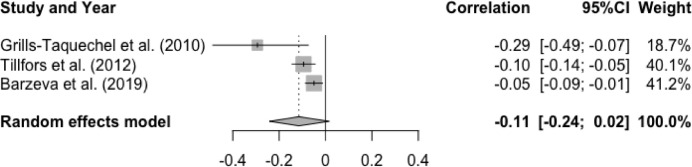
Forest Plot of Correlations between T1 Peer Acceptance and T2 Social Anxiety and 95% Confidence Interval for Random Effects Model

The mean effect size for the meta-analysis examining the association between T1 peer acceptance and T2 social anxiety was statistically non-significant, r = -.11, p = .10, 95% CI [-0.24, 0.02], suggesting that higher levels of peer acceptance at baseline were not associated with lower levels of prospective social anxiety (See Figure 4). There was a significant and moderate degree of heterogeneity, Q(2) = 6.10, p < .05, I^2^ = 67.2%. None of the moderator effect was statistically significant: age (Q(1) = 0.02, p = .90), gender (Q(1) = 0.58, p = .45), interval (Q(1) = 0.08, p = .78), and publication year (Q(1) = 1.06, p = .30).

#### 3.3.3 Peer rejection

**Figure 5:**
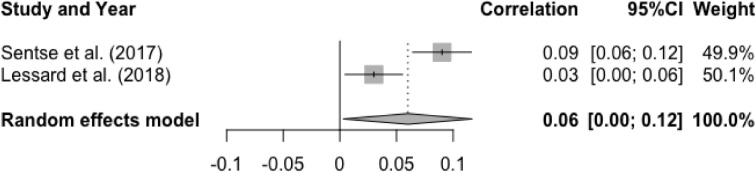
Forest Plot of Correlations between T1 Peer Rejection and T2 Social Anxiety and 95% Confidence Interval for Random Effects Model

For studies of peer rejection, the mean effect size for the association between baseline peer rejection and later social anxiety was statistically significant, r = .06, p < .05, 95% CI [0.005, 0.12], suggesting that higher levels of peer rejection at baseline were associated with higher levels of prospective social anxiety (See Figure 5). Heterogeneity test was significant and substantial, Q(1) = 10.60, p < .01, I^2^ = 90.6%. Moderator analyses were not conducted due to small sample size (n = 2).

#### 3.3.4 Peer victimization

**Figure 6:**
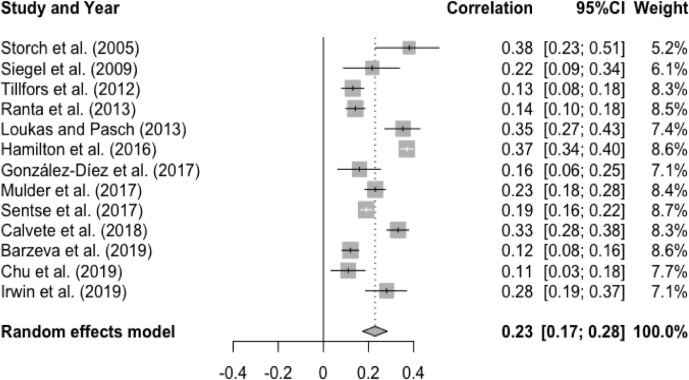
Forest Plot of Correlations between T1 Peer Victimization and T2 Social Anxiety and 95% Confidence Interval for Random Effects Model

There was a significant and small mean effect size among studies that examined the association between T1 peer victimization and T2 social anxiety, r = .23, p < .0001, 95% CI [0.17, 0.28]. This result suggests that higher levels of peer victimization at baseline were associated with higher levels of social anxiety at follow-up (See Figure 6). Heterogeneity was statistically significant and substantial, Q(12) = 198.47, p < .0001, I^2^ = 94.0%. However, there was a non-significant moderator effect of age (Q(1) = 0.63, p = .43), gender (Q(1) = 0.93, p = .34), interval (Q(1) = 0.10, p = .76), and publication year (Q(1) = 0.59, p = .44).

### 3.4 Meta-analyses of T1 Social Anxiety and T2 Peer Functioning Data

#### 3.4.1 Friendship quality

**Figure 7:**
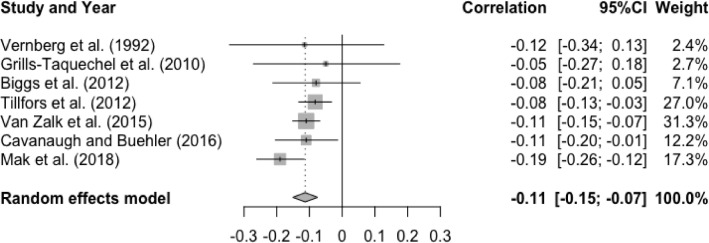
Forest Plot of Correlations between T1 Social Anxiety and T2 Friendship Quality and 95% Confidence Interval for Random Effects Model

The mean effect size for the meta-analysis examining the association between T1 social anxiety and T2 friendship quality was statistically significant and small, r = -.11, p < .0001, 95% CI [-0.15, -0.07], suggesting that higher levels of social anxiety at baseline were associated with lower levels of friendship quality at follow-up (See Figure 7). Heterogeneity test was statistically non-significant, Q(6) = 6.20, p = .40, I^2^ = 3.2%.

**Figure 8:**
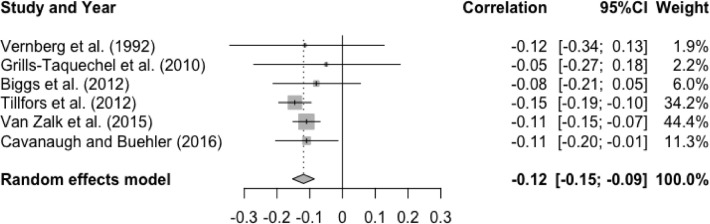
Forest Plot of Correlations between T1 Social Anxiety and T2 Positive Friendship Quality and 95% Confidence Interval for Random Effects Model

When examining studies of positive friendship quality alone (n = 3), the mean effect size for the association between T1 social anxiety and T2 positive friendship quality was small and statistically significant, r = -.12, p < .0001, 95% CI [-0.15, -0.09]. These results suggested higher levels of social anxiety at baseline were associated with lower levels of positive friendship quality at follow-up (See Figure 8). Heterogeneity test was statistically non-significant, Q(5) = 2.02, p = .85, I^2^ = 0%. Meta-analysis was not conducted for negative friendship quality because effect size data was reported in one study (Tillfors et al., 2012).

#### 3.4.2 Peer acceptance

**Figure 9:**
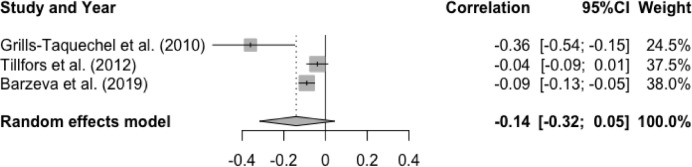
Forest Plot of Correlations between T1 Social Anxiety and T2 Peer Acceptance and 95% Confidence Interval for Random Effects Model

In studies examining the association between T1 social anxiety and T2 peer acceptance, meta-analysis showed a non-significant mean effect size for the association between T1 social anxiety and T2 peer acceptance, r = -.14, p = .13, 95% CI [-0.32, 0.05]. These results indicated that higher levels of social anxiety at baseline were not associated with lower levels of peer acceptance at follow-up (See Figure 9). Of note, there was a significant and high heterogeneity, Q(2) = 9.28, p < .01, I^2^ = 78.5%. None of the moderator effect was statistically significant: age (Q(1) = 0.32, p = .57), gender (Q(1) = 2.36, p = .12), interval (Q(1) = 0.57, p = .45), and publication year (Q(1) = 0.37, p = .54).

#### 3.4.3 Peer rejection

**Figure 10:**
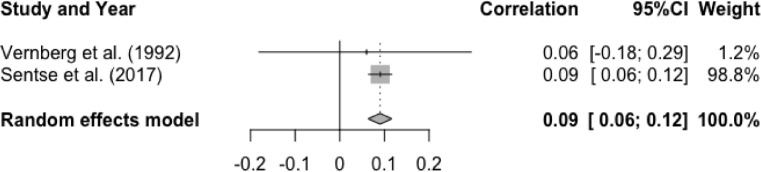
Forest Plot of Correlations between T1 Social Anxiety and T2 Peer Rejection and 95% Confidence Interval for Random Effects Model

There was a significant mean effect size for the association between T1 social anxiety and T2 peer rejection, r = .09, p < .0001, 95% CI [0.06, 0.12]. These results indicated that higher levels of baseline social anxiety were associated with higher levels of peer rejection at follow-up (See Figure 10). Heterogeneity test was non-significant, Q(1) = 0.06, p = .81, I^2^ = 0%. Moderator analysis was not performed due to small number of studies (n = 2).

#### 3.4.4 Peer victimization

**Figure 11:**
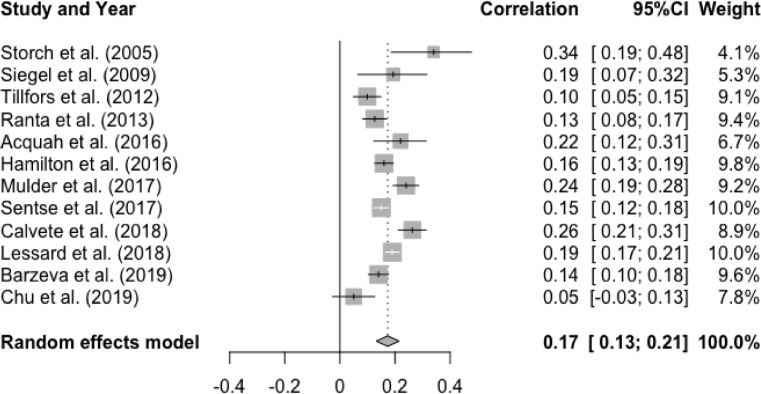
Forest Plot of Correlations between T1 Social Anxiety and T2 Peer Victimization and 95% Confidence Interval for Random Effects Meta-Analysis Model

The mean effect size for the meta-analysis examining the association between T1 social anxiety and T2 peer victimization was statistically significant and small, r = .17, p < .0001, 95% CI [0.13, 0.21]. This result suggests higher levels of social anxiety at baseline were associated with higher levels of peer victimization at follow-up (See Figure 11). Heterogeneity was statistically significant and substantial, Q(12) = 57.25, p < .0001, I^2^ = 79.0%. However, there was no significant moderator effect of age (Q(1) = 0.39, p = .53), gender (Q(1) = 3.09, p = .08), interval (Q(1) = 0.59, p = .44), and publication year (Q(1) = 0.003, p = .96).

### 3.6 Publication bias

There was a lack of asymmetry in funnel plots involving friendship quality, peer acceptance, and peer rejection (see Supplementary Materials, Appendix 3). A degree of asymmetry for peer victimization was found, as studies with a small standard error tended to scatter outside the funnel plot. However, in each meta-analysis, the Egger's regression intercept was statistically non-significant (ps > .05), indicating there was no substantial publication bias.

## 4 Discussion

### 4.1 Summary of findings

This review aimed to synthesize findings on the prospective associations between peer functioning and social anxiety in adolescents. Meta-analyses of 23 studies showed that friendship quality, peer rejection, and peer victimization were each prospectively associated with social anxiety, and social anxiety was also associated with later peer functioning in these domains. Peer acceptance was not associated with later social anxiety. Social anxiety was not associated with later peer acceptance. Average age of participants was found to moderate the association between baseline friendship quality and later social anxiety. Findings therefore suggest reciprocal links between peer functioning and social anxiety in adolescents.

Consistent with the findings reported by Epkins and Heckler (2011), there is evidence to suggest that adolescents with higher levels of friendship quality tend to experience lower levels of social anxiety over time. Positive peer relationships appear to buffer against the development of social anxiety. One possible mechanism is via social beliefs; specifically, cognitive behavioral models suggest cognitions are central to social anxiety (Clark & Wells, 1995; Heimberg et al., 2010; Rapee & Heimberg, 1997), and it may be that positive peer interactions serve as evidence for positive (e.g. “I am acceptable”) and against negative (e.g. “I am unlikable”) social beliefs about the self.

In terms of the magnitude of effect size, peer victimization is more strongly related to prospective social anxiety compared to friendship quality, peer rejection, and peer acceptance (correlation coefficients of .23; .11; .06; .11 respectively). In line with cognitive models of social anxiety (Clark & Wells, 1995; Rapee & Heimberg, 1997), peer victimization may inform or confirm one's negative beliefs about one's social acceptability and generate social anxiety. For example, an individual who is excluded by their peers may take this as evidence that they are unlikeable, and then feel more anxious about rejection in the future as a result. Our findings are also consistent with proposals of Wong and Rapee (2016), who suggested that peer relations that involve negative evaluation (i.e. peer victimization) are likely to be most influential in the development and maintenance of social anxiety.

The negative association between social anxiety and later friendship quality, and the positive association between social anxiety and later peer rejection suggest high levels of social anxiety may have a deleterious effect on adolescent relationships. One possible explanation is socially anxious youth may behave in ways that prevent them from forming or maintaining positive peer relationships (Biggs et al., 2012). For example, socially anxious youth may avoid asking questions and so appear socially disinterested, which may trigger negative responses from their peers (Leigh & Clark, 2018).

Unlike the other three constructs, our analysis indicated peer acceptance did not have a bidirectional relationship with social anxiety. This finding should be interpreted with caution as this analysis may have been underpowered to detect significant differences due to small sample size (n = 3). Furthermore, there is substantial heterogeneity across studies. The presence of high heterogeneity may be explained by the use of different measures for peer acceptance or the presence of other peer constructs.

Moderator analysis suggests high quality friendship may offer a greater protective effect against social anxiety in younger adolescents than older adolescents. Variations in study design, including the proportion of female participants, time interval, and publication year, do not seem to explain the significant heterogeneity in study outcomes. The current review included studies that examined adolescents aged 11–19 years. Young people typically transition to secondary school at the age of 11, and therefore one interpretation of the current finding is that the peer environment and formation of peer bonds is particularly important at this time; having friends who are caring and supportive may reinforce the social belief that they are accepted by peers and therefore reduce the likelihood of developing social anxiety. Consistent with this finding, a recent meta-analysis has found that the associations between some aspects of friendship quality and depressive symptoms are stronger for younger than older youth (Schwartz-Mette et al., 2020).

The interrelationships between peer-related factors may help explain some of the bidirectional relationships reported in this review. There is evidence suggesting that rejected children were more likely to experience peer victimization over time (Demol et al., 2020), and therefore peer victimization may partially mediate the impact of peer rejection on social anxiety, creating a cascade effect. In addition, victimized children who have close friendships may be less likely to experience higher levels of social anxiety over time, as they have evidence to support the belief that they are liked or accepted by their peers. Therefore, positive friendship quality may buffer the impact of peer victimization on social anxiety. Further research is needed to study these complex interactions by considering some of the recent research findings (e.g. Freitas et al., 2019; Schacter et al., 2019).

### 4.2 Limitations

There are several limitations to our study. First, peer functioning has been assessed using a variety of methods and there is no single way to categorize aspects of peer functioning. Results may differ if studies were categorized differently. For example, the moderator effect of age on the association between T1 friendship quality and T2 social anxiety was not statistically significant after excluding studies of negative friendship quality. Second, most of the studies in this review used self-report measures to assess peer functioning and social anxiety. This research design may introduce shared-method variance and inflate the association between peer functioning and social anxiety. Third, significant heterogeneity was observed in our meta-analyses involving peer acceptance, peer rejection, and peer victimization. However, it was not possible to examine other potential moderators, such as the use of self-report (versus peer-report) measures and aspects of peer functioning, due to the lack of studies in each subcategory. Fourth, only one study was conducted with a clinical sample and therefore the present results may not generalize to clinical samples. Fifth, there were not enough studies to conduct a meta-analysis for negative friendship quality, and further study is needed. Finally, depressive symptoms were not controlled for in the meta-analyses. Depressive symptoms are associated with aspects of friendship (Schwartz-Mette et al., 2020) and tend to co-occur with social anxiety (Melton et al., 2016). Therefore, these symptoms may partially account for the relationships between social anxiety and peer functioning reported in this study.

### 4.3 Implications

Notwithstanding these limitations, this review is the first of its kind to examine prospective studies on the direction of effects between peer functioning and social anxiety in adolescents. The strengths of this review include a consideration of different dimensions of peer functioning, the inclusion of prospective studies, and a quantitative assessment of effect sizes. These methodological strengths have enabled us to infer whether there is a reciprocal link between peer functioning and social anxiety in adolescents.

Environmental factors, specifically peer relations, are relevant to the development or maintenance of adolescent social anxiety. To extend our understanding of the impact of interpersonal factors on the development of social anxiety, measurement other than self-report questionnaires should be used more often in future research. For instance, peer nomination can be used to assess peer acceptance. Observations reported by parents and teachers may serve as indirect measures for peer victimization. Given there is evidence of a reciprocal association between peer functioning and social anxiety, it may be helpful to understand underlying mechanisms and identify modifiable mediators. Some mediators may be related to how adolescents respond to social situations when they feel anxious. For example, avoidance safety behaviors may evoke negative responses from others and reinforce social fears, and thus these behaviors may mediate the impact of social anxiety on peer relationships. Peer-related factors, such as peer victimization, may also mediate the impact of peer rejection on social anxiety in adolescents. To improve our understanding on the interrelationships between peer-related factors and their joint influence on social anxiety, research is needed to measure multiple aspects of peer functioning at different time points.

Our results suggest peer functioning and social anxiety are viable targets for early psychological prevention and intervention. To reduce youth's risk of developing social anxiety, schools may apply strategies to strengthen peer relationships. For example, teaching students social and emotional skills, using buddying systems to support students who are settling into a new school, and supporting students to negotiate interpersonal conflicts. Importantly, targeting peer victimization should be made a priority as it has the strongest bidirectional association with social anxiety compared to other dimensions of peer functioning. School-based intervention targeting relational and reputational peer victimization could be a potentially helpful approach in preventing the development of social anxiety and depression in adolescents (e.g. La Greca et al., 2016). Individual psychological interventions focusing on intra- and inter-personal processes can also be helpful. For example, in Cognitive Therapy for Social Anxiety Disorder in Adolescents (CT-SAD-A; Leigh & Clark, 2016), young people can learn to identify and stop behaviours that may elicit negative reactions from their peers. CT-SAD-A can be augmented by working with teachers, for example, planning school-based behavioral experiments with teachers.

## 5 Conclusions

This review examined evidence of prospective associations between peer relations and social anxiety in adolescents. We found a bidirectional, prospective association between aspects of peer functioning (i.e. friendship quality, peer rejection, and peer victimization) and social anxiety. The protective effect of friendship quality on later social anxiety was stronger in younger adolescents than older adolescents. The moderator effects of gender, time interval, and publication year were statistically non-significant. Further research is needed to examine these bidirectional associations using informant measures for peer relations and social anxiety, and findings need to be replicated and extended to a clinical sample.

## Author Statement

Kenny Chiu: Investigation, Project administration, Formal analysis, Software, Writing - Original draft. David M Clark: Conceptualisation, Methodology, Supervision, Writing - reviewing & editing. Eleanor Leigh: Conceptualisation, Methodology, Supervision, Investigation, Project administration, Writing - reviewing & editing.

## Role of the funding source

The funders had no involvement in study design; collection, analysis and interpretation of data; writing of the report; or in the decision to submit the article for publication.

## Declaration of Competing Interest

The authors declare that they have no known competing financial interests or personal relationships that could have appeared to influence the work reported in this paper.

## Appendix Supplementary materials

Image, application 1
